# Use of Machine Perfusion to Increase the Number of Expanded Criteria Deceased Donor Kidney Transplants: A Pharmacoeconomic Analysis

**DOI:** 10.1097/TXD.0000000000001668

**Published:** 2024-07-05

**Authors:** Helio Tedesco Silva, Teresa Raquel de Moraes Ramos, Deise De Boni Monteiro de Carvalho, Gustavo Fernandes Ferreira, João Marcelo Medeiros de Andrade, Luis Gustavo Modelli de Andrade, Mario Abbud-Filho, Renato Demarchi Foresto, Roberto Ceratti Manfro, Ronaldo de Matos Esmeraldo, Tainá Veras de Sandes Freitas, Valter Duro Garcia, José Medina Pestana, Marcelo Cunio Machado Fonseca

**Affiliations:** 1 Hospital do Rim, Fundação Oswaldo Ramos, São Paulo, Brazil.; 2 Disciplina de Nefrologia, Escola Paulista de Medicina, UNIFESP, São Paulo, Brazil.; 3 Women’s Health Technology Assessment Center, Department of Gynecology, Federal University of São Paulo, Medical School, São Paulo, Brazil.; 4 Axia.Bio Life Sciences, São Paulo, Brazil.; 5 Axia.Bio Life Sciences, Miami, FL.; 6 Hospital São Francisco na Providência de Deus, Rio de Janeiro, Brazil.; 7 Transplant Unit Santa Casa Juiz de Fora, Juiz de Fora, Brazil.; 8 Unidade de Transplantes—Instituto de Medicina Integral Prof. Fernando Filgueira, Recife, Brazil.; 9 Universidade Estadual Paulista, Botucatu, Brazil.; 10 Hospital de Base, Medical School FAMERP, São José do Rio Preto, Brazil.; 11 Hospital de Clínicas de Porto Alegre, Porto Alegre, Brazil.; 12 Hospital Geral de Fortaleza, Fortaleza, Brazil.; 13 Santa Casa de Misericórdia de Porto Alegre, Porto Alegre, Brazil.

## Abstract

**Background.:**

The discard of expanded criteria donor (ECD) kidneys is unacceptably high, considering the growing demand for transplantation. Using machine perfusion may reduce the discard rate, increase the number of transplants, and reduce mortality on the waiting list.

**Methods.:**

We developed a 5-y Markov model to simulate incorporating the pulsatile perfusion machine into the current government-funded healthcare system. The model compared the universal use of static cold storage for all kidneys with the selective use of machine perfusion for ECD kidneys. Real-life data were used to compose the cohort characteristics in this model. This pharmacoeconomic analysis aimed to determine the cost-effectiveness and budgetary impact of using machine perfusion to preserve ECD kidneys.

**Results.:**

Compared with the universal use of static cold storage, the use of machine perfusion for ECD kidneys was associated with an increase in the number of kidney transplants (n = 1123), a decrease in the number of patients on the waiting list (n = 815), and decrease in mortality (n = 120), with a cost difference of US dollar 4 486  009 in the period. The budget impact analysis revealed an additional cost of US dollar 4 453 749 >5 y. The budget impact analysis demonstrated a progressive reduction in costs, becoming cost-saving during the last year of the analysis.

**Conclusions.:**

This stochastic model showed that incorporating machine perfusion for ECD kidneys is most often a dominant or cost-effective technology associated with an increase in the number of transplants and a reduction in the number of patients on the waiting list, reducing mortality on the waiting list.

Chronic kidney disease (CKD) poses a significant global public health challenge, as its prevalence continues to rise because of the increasing occurrence of chronic conditions like diabetes and hypertension, coupled with extended life expectancies. Patients with end-stage CKD require effective treatment options, with kidney transplantation emerging as a superior alternative to dialysis. Transplantation offers better outcomes, including a decreased risk of cardiovascular events, lower mortality rates, and improved quality of life, all at a lower long-term cost than dialysis.^1,2^

Despite the benefits of transplantation, the challenge lies in the utilization of kidneys from expanded criteria donors (ECDs). The increased use of ECD kidneys worldwide seeks to address the organ supply demand gap, yet logistical challenges and higher discard rates persist.^3^ Innovative strategies, particularly adopting hypothermic machine perfusion (HMP) systems, show promise in improving the utilization and outcomes of ECD kidney transplants. Recent studies suggest that HMP reduces delayed graft function (DGF) rates and enhances graft survival compared with static cold storage (SCS).^4,5^

In our country, the mean proportion of transplants with ECD kidneys is around 26.8%, and the use of SCS remains predominant despite the high incidence of DGF of 54%, ranging from 29.9% to 87.7%.^6,7^ A national trial demonstrated that machine perfusion significantly reduced DGF incidence, albeit with cost considerations.^8,9^ In the context of limited resources and budget constraints, a pharmacoeconomic analysis explores the cost-effectiveness (CE) of implementing HMP specifically for preserving kidneys recovered from ECD. The hypothesis proposes that restricting HMP to ECD kidneys could be a strategic and cost-effective approach by primarily reducing the discard rate, potentially leading to increased transplant rates, reduced waitlist numbers, and decreased overall mortality. Therefore, this pharmacoeconomic analysis evaluates the CE and budgetary impact of incorporating HMP for preserving kidneys recovered from ECD.

## MATERIALS AND METHODS

We developed a Markov model built from the perspective of the public payer that uses real-life clinical and cost data extracted from local databases, the Brazilian Transplantation Registry, the Brazilian Government Health Information System, particularly the Table of Procedures, Medications, Orthotics, Prostheses, and Special Materials (Sistema de Gerenciamento da Tabela de Procedimentos, Medicamentos, Orteses, Proteses e Materiais Especiais [SIGTAP]), and the Brazilian Institute of Geography and Statistics. It is essential to emphasize that most procedure costs in the government table have been unchanged for many years. Only hemodialysis had an adjustment in the costs required for the model.

The model does not assume any changes in the incidence of DGF or an increase in graft survival associated with using machine perfusion. Patient and graft survival data were extracted from a meta-analysis.^3^

### Population

Real-world data were used to compose the cohort characteristics in this model. The population simulated in the model consisted of Brazilian adult patients with CKD on the waiting list for a deceased donor kidney transplant in December 2021.^10^ For this analysis, we used the standard definition for ECD (>60 y or 50–59 y with at least 2 of the following criteria: hypertension, cerebrovascular cause of brain death, and terminal serum creatinine level > 1.5 mg/dL).^11^

### Structure of the Model

**FIGURE 1.**
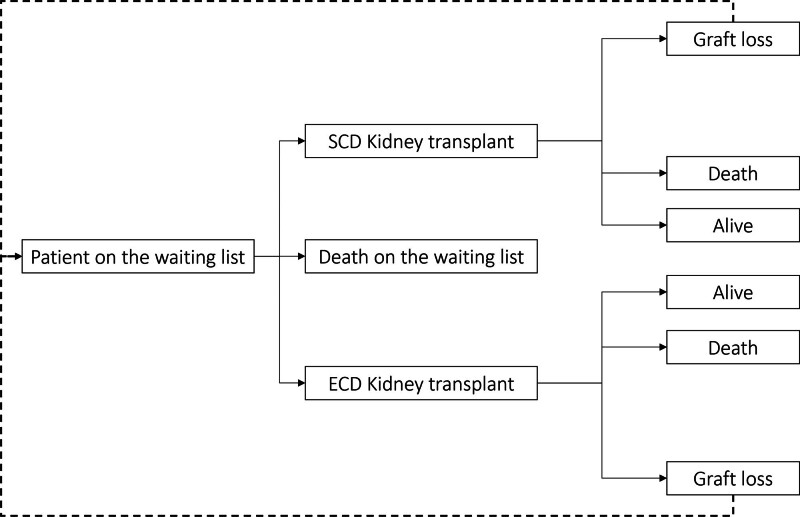
Markov model. ECD, expanded criteria donor; SCD, standard criteria donor.

We used the Markov model to simulate 2 cohorts of individuals on the waiting list for kidney transplantation (Figure 1). To input the proportion of standard criteria donor (SCD) and ECD offers in the model, we extracted data from the largest donor service area in the country (São Paulo metropolitan area—21 million inhabitants) with the highest transplant activity. In 2021, there were 1586 kidney recovered, 1279 (81%) from SCD and 307 (19%) were from ECD. The number of SCD transplanted was 1127, 88% of the recovered organs, whereas the number of ECD kidney transplants was 191, corresponding to 62% of the offers. Therefore, in the first cohort of the model, where all kidneys were preserved using SCS, we estimated that 88% of SCD and only 62% of the ECD kidneys offered were utilized.^12^ In the second cohort of the model, SCD kidneys were still preserved using SCS, and 88% of them were transplanted. On the other hand, all ECD kidneys were preserved using the perfusion machine and 88% of them were transplanted.

Patients on the waiting list may die or receive a kidney transplant in both cohorts. After receiving a kidney transplant, patients may remain healthy with a functioning graft, die with a functioning graft, or lose the graft, returning to the waiting list regardless of the donor type, SCD, or ECD.

The model had a 1-y cycle time. A half-cycle adjustment was applied to all health expenditures and benefits. Every year, a new cohort of people needing kidney transplants was added to the model. The analysis was conducted from the perspective of the leading healthcare provider, the government’s public healthcare system (Sistema Único de Saúde [SUS]). The health outcomes considered in the model were death, number of transplants performed, and avoided dependence on dialysis.

### Model Inputs

#### Waiting List and Transplants

In December 2021, 27 613 patients were on the waiting list to receive a deceased donor kidney transplant in Brazil.^10^ Based on data from the Brazilian Transplantation Registry from 2015 to 2020, the number of patients added to the transplant waiting list and the annual number of kidney transplants were forecasted for the next 5 y, the time horizon (Table 1).^10^

**TABLE 1.** Model inputs

Year	Patients added to the waiting list	Deceased donor kidney transplants
Year 1	9949	4994
Year 2	16 132	5117
Year 3	10 913	5242
Year 4	17 096	5370
Year 5	11 877	5502

The predicted annual number of patients added to the waiting list and the number of kidney transplants are based on the 2021 “Registro Brasileiro de Transplante” published by “Associação Brasileira de Transplante de Órgãos.”^10^

There is no national data on mortality on the waiting list. Therefore, the annual probability of dying while on the waiting list, 8.08%, was based on data from 12 451 patients on the waiting list of the Hospital do Rim, São Paulo, Brazil, a high-volume kidney transplant center in 2021.

#### Survival

A recent systematic review and meta-analysis published the 5-y patient mortality and graft loss rates by donor type.^3^ The annual probabilities used in the model were retrieved using Engauge Digitizer software from pooled Kaplan-Meier curves for death-censored graft survival and patient survival (**Table S1, SDC**, http://links.lww.com/TXD/A669). We also considered general population mortality from the 2020 Brazilian Institute of Geography and Statistics.

#### Costs

All costs are provided in US dollar (USD) using the 2021 average dollar value (1 USD = R$ 5.39—https://zebracambio.com.br/media-do-dolar-em-2021/). All procedures and costs associated with the extraction, cold static preservation, and transportation of the kidneys were considered according to the SIGTAP (**Table S2, SDC**, http://links.lww.com/TXD/A669).^13^ The costs associated with using HMP, including training, are in **Table S3 (SDC**, http://links.lww.com/TXD/A669).

The deceased donor kidney transplant procedure associated costs, obtained from the 2019 Hospital Information System database, including the dialysis section the DGF period, were USD 8254.39.^14^ The annual cost of monitoring a kidney transplant recipient was USD 2715.60.^9^

Dialysis costs were calculated using the microcosting provided by Gouveia et al.^2^ The figures were compared with those in the current SIGTAP table, and the hemodialysis reimbursement value used by Gouveia et al^2^ was revised to USD 40.53 (**Table S4**, **SDC**, http://links.lww.com/TXD/A669).

### Discount

The 5% discount rate was used for economic outcomes in the CE model’s base case. The univariate and probabilistic sensitivity analyses used a discount rate of 0%–10%. In the budget impact analysis (BIA), no discount was used.^14^

### Sensitivity Analysis

Sensitivity analysis, which can be deterministic or probabilistic, was used to investigate parameter uncertainty. The main distinction between the two was in the method of expressing parameter variation. A set of values represents the plausibility of parameter variation in the deterministic sensitivity analysis. Random distributions were utilized instead of specific values in the parameter variation in the probabilistic sensitivity analysis. Therefore, 1-way deterministic sensitivity analysis and a second-order Monte Carlo simulation with 1000 iterations were carried out. The outcomes were plotted on a tornado diagram and CE plan. The parameters used in the deterministic and probabilistic sensitivity analysis are shown in **Table S5 (SDC**, http://links.lww.com/TXD/A669).

Brazil has no official CE threshold or consistent CKD quality-adjusted life year assessment. As a result, we could not estimate the willingness to pay for the perfusion machine per quality-adjusted life year.^15^ For liberality, marking, and dimensioning purposes, we used a CE threshold of USD 19 570.52, corresponding to 3 gross domestic product (GDP) per capita.

## RESULTS

### Case-base Scenario

According to the model, compared with the cold static organ preservation, the use of machine perfusion for ECD kidneys was associated with an increase of 1123 kidney transplants, 815 fewer patients remaining on dialysis, and 120 fewer deaths, at a cost difference of USD 4 486 009 (Table 2). The increase in follow-up costs was attributable to the increased number of patients living with a functioning graft (**Figure S1, SDC**, http://links.lww.com/TXD/A669).

**TABLE 2.** Main outcomes of the cost-effectiveness model

Outcomes	Machine perfusion	Cold storage	Difference	ICER
Patients alive (n)	54.311	54.191	120	37 393
Performed transplants (n)	20.008	18.884	1.123	3994
Dialysis dependent (n)	36.445	37.260	815	5505
Total cost (USD)	1 570 902 471	1 566 416 462	4 486 009	

ICER, incremental cost-effectiveness ratio; USD, US dollar.

### One-way Sensitivity Analyses

The percentage of transplants from standard donors in the machine perfusion cohort was the parameter that most affected the model in all outcomes, CE for transplants performed, CE for dialysis avoided, and CE for death avoided (**Tables S6–S8**, **SDC**, http://links.lww.com/TXD/A669).

### Probabilistic Sensitivity Analyses

The average CE per transplant performed after 1000 iterations was USD 3597. Twenty-five percent of iterations were dominant (second quadrant), 40.0% were cost-effective (first quadrant below the 3 GDPs/capita threshold), and 18.4% were below the 1 GDP/capita threshold (**Figure S2, SDC**, http://links.lww.com/TXD/A669).

The average CE per dialysis avoided after 1000 iterations was USD 4867. Forty-two percent of the iterations were dominant (second quadrant), while 17.8% were cost-effective (first quadrant below the 3 GDPs/capita threshold), and 11.0% fell below the 1 GDP/capita threshold (**Figure S3, SDC**, http://links.lww.com/TXD/A669).

The average CE per death avoided after 1000 iterations was USD 33 702 (**Figure S4, SDC**, http://links.lww.com/TXD/A669).

### Budget Impact Analysis

Over 5 y, this analysis revealed an additional cost of USD 4 453 749 (Table 3). Notably, the BIA demonstrated progressive cost reduction, becoming cost-saving during the last year of the analysis.

**TABLE 3.** Budget impact >5 y of incorporating pulsatile perfusion machines into SUS

Scenarios	2023	2024	2025	2026	2027	In 5 y
Current scenario	248 222 681	306 552 652	360 857 778	413 840 093	458 761 123	1 788 234 327
Static organ preservation (SCD/ECD)						
Proposed scenario	250 614 032	308 369 890	361 821 148	414 043 294	457 839 712	1 792 688 076
Static organ preservation (SCD)						
Machine perfusion (ECD)						
Budget impact	2 391 351	1 817 238	963 371	203 201	–921 411	4 453 749

All values are in USD.

ECD, expanded criteria donor; SCD, standard criteria donor; SUS, Sistema Único de Saúde; USD, US dollar.

## DISCUSSION

After 5 y of follow-up, this CE study found that using machine perfusion for kidneys from ECD and static organ preservation for SCD together led to an increase of 1123 kidney transplants, a decrease of 815 patients on dialysis, and a decrease of 120 deaths. These positive outcomes were observed, along with an additional cost of USD 4 453 749 revealed in the BIA for the same period. It is worth noting that the BIA demonstrates a steady decline in impact during the initial 4 y, ultimately reaching a negative value in the fifth year, hence resulting in resource savings for the system. This benefit derives primarily from a marked reduction in patients requiring dialysis, underscoring this innovative strategy’s dominance over universal static cold preservation.

The predominant factor influencing the model’s results was the proportion of kidney transplants from ECD in the cohort where a perfusion machine was not used. Notably, increasing the utilization of ECD kidneys beyond the 50% threshold remained a cost-effective strategy, yielding benefits in terms of reduced number of patients on dialysis and increased number of transplants. This aligns with the consistent findings from related studies, reinforcing the economic viability of this approach.^16,17^

The growing use of machine perfusion for kidney preservation is supported by numerous studies indicating its association with a reduced incidence of DGF and superior graft function or survival compared with SCS.^4,16,18,19^ The selective use of machine perfusion exclusively to ECD kidneys remains a subject of ongoing debate, suggesting that machine perfusion cost-effectively delivers significant clinical benefits across all donor kidney types.^20^ Moreover, this selective approach holds particular significance for our national transplant program, given the observed high incidence of DGF, ranging between 50% and 60%.^6^ Within this scenario, a previous multicenter study demonstrated a significant reduction in the incidence of DGF, from 61% to 45%, associated with using machine perfusion.^8^ However, while conservative, this pharmacoeconomic analysis primarily focused on the potential decrease in the discard rate. First, machine perfusion offers a promising strategy for reevaluating kidneys initially deemed unsuitable for transplantation, mitigating the discard rate. Secondly, the model did not account for the potential reduction in the incidence of DGF and improved long-term graft survival, which remains debatable.

This pharmacoeconomic modeling comes with inherent limitations associated with the specific characteristics of our public healthcare system, coverages, and reimbursements. Kidneys preserved with machine perfusion but ultimately discarded based on functional parameters may yield inferior outcomes. Moreover, in line with a recent meta-analysis, the model does not incorporate any potential increase in long-term graft survival associated with kidneys preserved using machine perfusion. Finally, the allocation of kidneys from ECD poses a growing challenge because of the higher risks of adverse outcomes related to these organs, a consideration not included in this analysis. Consequently, the findings may not readily apply or be extrapolated to other transplant centers with different healthcare systems and patient demographic characteristics.

In summary, the assumptions embedded in this pharmacoeconomic model suggest that incorporating machine perfusion for preserving kidneys from ECD is associated with an augmentation in the number of transplants performed, a reduction in the number of patients on dialysis, and a decline in mortality rate with a reasonable cost per dialysis avoided and additional transplant performed. Therefore, this strategic approach is not only cost-effective but also demonstrates a favorable impact on the budget, which is crucial for our public health system operating within limited resources.

## Supplementary Material


