# Supplementary material for: Use of Machine Perfusion to Increase the Number of Expanded Criteria Deceased Donor Kidney Transplants: A Pharmacoeconomic Analysis
Source: Transplant Direct. 2024 Jul 5;10(8):e1668. doi: 10.1097/TXD.0000000000001668 (PMC11230806; doi:10.1097/TXD.0000000000001668)
Supplement: Supplementary file 1 [file txd-10-e1668-s001.pdf]

Table S1. Annual probabilities of patient death and graft loss after kidney transplantation.

Year after kidney transplantation	Death		Death-censored graft loss	
	ECD	SCD	ECD	SCD
1	7.14%	4.11%	14.98%	6.62%
2	4.23%	2.42%	7.37%	5.18%
3	3.82%	2.67%	7.50%	5.27%
4	4.38%	2.94%	9.09%	5.98%
5	4.15%	2.42%	10.00%	4.11%

Adapted from the systematic review and meta-analysis[17].
ECD: expanded criteria donor; SCD: standard criteria donor.

Table S2. Costs of recovery and transportation of deceased donor kidneys**.**

**Preparation**	**Cost (USD)**
Evaluation of potential deceased organ and tissue donors for transplantation	40
Clinical evaluation of brain death in children over 2 years	40
ABO blood typing in potential organ donors	3
Complementary exams for brain death diagnosis	111
Daily ICU cost of a potential organ donor	94
**Interview**	
Interview with the family of a brain-dead potential organ donor in preparation for organ donation	78
**Surgery Center**	
Hemodynamic management of the possible donor and cost of the operating room for organ recovery	167
Coordination of the operating room for organ and tissue recovering for transplantation	74
**Recovery**	
Unilateral or bilateral kidney extraction for transplant - deceased donor	217
Kidney preservation solution	65
**Effective donors**	
Number of organs recovered and transplanted organs: maximum of 06 per donor	48
Interstate displacement of professional staff for organ recovery	167
**Total cost**	**1,104**

Table S3. Cost of using the machine perfusion according to manufacturer's pricing information.

**Parameters**	**Cost (USD)**
Acquisition, per machine	16,000
Operation, per kidney	2,252
Perfusion Circuit	2,000
KPS1 perfusion fluid	180
Disposable cannula	60
Recovery of the deceased donor kidneys*	1,040

*Estimated microcosting described in Table Supplemental Table 2, excluding the cost of the preservation solution.

Table S4. Annual hemodialysis microcosting per patient per year.

**Parameters**	**Annual cost (USD)**
Hemodialysis (maximum of 3 sessions per week)	5,837
Exams	100
Venous access	152
Drugs	1,453
Visits	22
Hospitalizations	219
**Total cost**	**7,783**

Table S5. Range of the parameters in the sensitivity analysis.

**Parameters**	**Mean**	**Distribution**	**Minimum**	**Maximum**
Mean age, years	50	Gama	40	60
Discount, %	0.05	Beta	0.00	0.10
Proportion of kidney transplants from ECD	0.19	Beta	0.17	0.21
Death on the waiting list	0.08	Beta	0.07	0.09
Machine perfusion cohort				
Kidney transplants from ECD	0.88	Beta	0.79	0.97
Kidney transplants from SCD	0.88	Beta	0.79	0.97
Cold storage cohort				
Kidney transplants from ECD	0.62	Beta	0.56	0.68
Kidney transplants from SCD	0.88	Beta	0.79	0.97
Machine perfusion preservation cost	3.292	Log-normal	2.962	3.621
Cold static preservation cost	1.105	Log-normal	994	1.215
Kidney transplant cost	8.254	Log-normal	7.429	9.080
Hemodialysis cost	7.782	Log-normal	7.004	8.561
Follow-up cost	2.716	Log-normal	2.444	2.987
Perfusion machine price	16.000	Log-normal	14.400	17.600
Number of machines purchased - Year 1	10	Gama	0	20
Number of machines purchased - Year 2	10	Gama	0	20
Number of machines purchased - Year 3	10	Gama	0	20
Number of machines purchased - Year 4	10	Gama	0	20
Number of machines purchased - Year 5	10	Gama	0	20

Assumption of ± 10%. All values are in USD.

Table S6. Univariate sensitivity analysis of the cost-effectiveness per transplant performed.

Parameter	Minimum Value	Average	Maximum Value	Variation
% of expanded criteria transplants without perfusion	-22,471	3,994	-804	47.857439%
% standard transplants without perfusion	-804	3,994	-22,471	47.857439%
% standard transplants employing perfusion	6,952	3,994	2,532	1.334539%
Dialysis cost	2,460	3,994	6,489	1.109246%
Transplant cost	5,984	3,994	2,004	1.082475%
Perfusion operational cost	2,880	3,994	5,108	0.339227%
% expanded criteria transplants employing perfusion	3,169	3,994	4,820	0.186217%
Follow-up cost	3,337	3,994	4,651	0.117924%
Discount	3,527	3,994	4,437	0.056474%
% death on the waiting list	4,258	3,994	3,731	0.018961%
Static organ preservation cost	3,740	3,994	4,244	0.017370%
Mean age	3,852	3,994	4,137	0.005546%
Number of machines purchased - Year 1	3,861	3,994	4,127	0.004817%
Number of machines purchased - Year 2	3,874	3,994	4,114	0.003944%
Number of machines purchased - Year 3	3,880	3,994	4,109	0.003577%
Number of machines purchased - Year 4	3,935	3,994	4,114	0.002186%
Perfusion machine price	4,057	3,994	3,943	0.000892%
Number of machines purchased - Year 5	3,938	3,994	4,051	0.000874%
% of organ with expanded criteria	3,938	3,994	4,050	0.000851%

All values are in USD.

Table S7. Univariate sensitivity analysis of the cost-effectiveness per avoided dialysis.

Parameter	Minimum Value	Average	Maximum Value	Variation
% of expanded criteria transplants without perfusion	-27,928	5,505	-1,087	47.348449%
Transplant cost	-1,087	5,505	-27,928	47.348449%
% standard transplants employing perfusion	9,582	5,505	3,490	1.571917%
Dialysis cost	3,391	5,505	8,945	1.306550%
% standard transplants without perfusion	8,249	5,505	2,762	1.275017%
Perfusion operational cost	3,970	5,505	7,041	0.399566%
% expanded criteria transplants employing perfusion	4,283	5,505	6,967	0.305316%
Follow-up cost	4,368	5,505	6,643	0.219340%
Discount	4,600	5,505	6,411	0.138900%
% death on the waiting list	5,060	5,505	5,961	0.034338%
Number of machines purchased - Year 4	5,869	5,505	5,142	0.022334%
Number of machines purchased - Year 3	5,309	5,505	5,702	0.006533%
Mean age	5,386	5,505	5,752	0.005675%
Static organ preservation cost	5,322	5,505	5,688	0.005674%
Number of machines purchased - Year 1	5,340	5,505	5,671	0.004646%
Number of machines purchased - Year 2	5,348	5,505	5,663	0.004214%
% of organ with expanded criteria	5,592	5,505	5,435	0.001051%
Number of machines purchased - Year 5	5,427	5,505	5,583	0.001030%
Perfusion machine price	5,429	5,505	5,582	0.001003%

All values are in USD.

Table S8. Univariate sensitivity analysis of the cost-effectiveness per death avoided.

	Minimum Value	Average	Maximum Value	Variation
% of expanded criteria transplants without perfusion	-82,690	37,393	-5,882	41.67811%
% standard transplants without perfusion	-5,882	37,393	-82,690	41.67811%
% standard transplants employing perfusion	65,083	37,393	23,707	4.94800%
Dialysis cost	23,030	37,393	60,751	4.11270%
Transplant cost	56,025	37,393	18,761	4.01344%
Perfusion operational cost	26,963	37,393	47,823	1.25774%
% expanded criteria transplants employing perfusion	29,088	37,393	47,323	0.96106%
Follow-up cost	29,665	37,393	45,121	0.69043%
Discount	31,243	37,393	43,543	0.43722%
Static organ preservation cost	39,859	37,393	34,927	0.07030%
Mean age	40,151	37,393	35,308	0.06780%
% death on the waiting list	36,059	37,393	38,727	0.02056%
Number of machines purchased - Year 1	36,150	37,393	38,636	0.01786%
Number of machines purchased - Year 2	36,268	37,393	38,518	0.01462%
Number of machines purchased - Year 3	36,322	37,393	38,464	0.01326%
Number of machines purchased - Year 4	36,808	37,393	38,582	0.00910%
Perfusion machine price	37,981	37,393	36,912	0.00331%
Number of machines purchased - Year 5	36,864	37,393	37,923	0.00324%
% of organ with expanded criteria	36,871	37,393	37,916	0.00316%

All values are in USD.

Figure S1

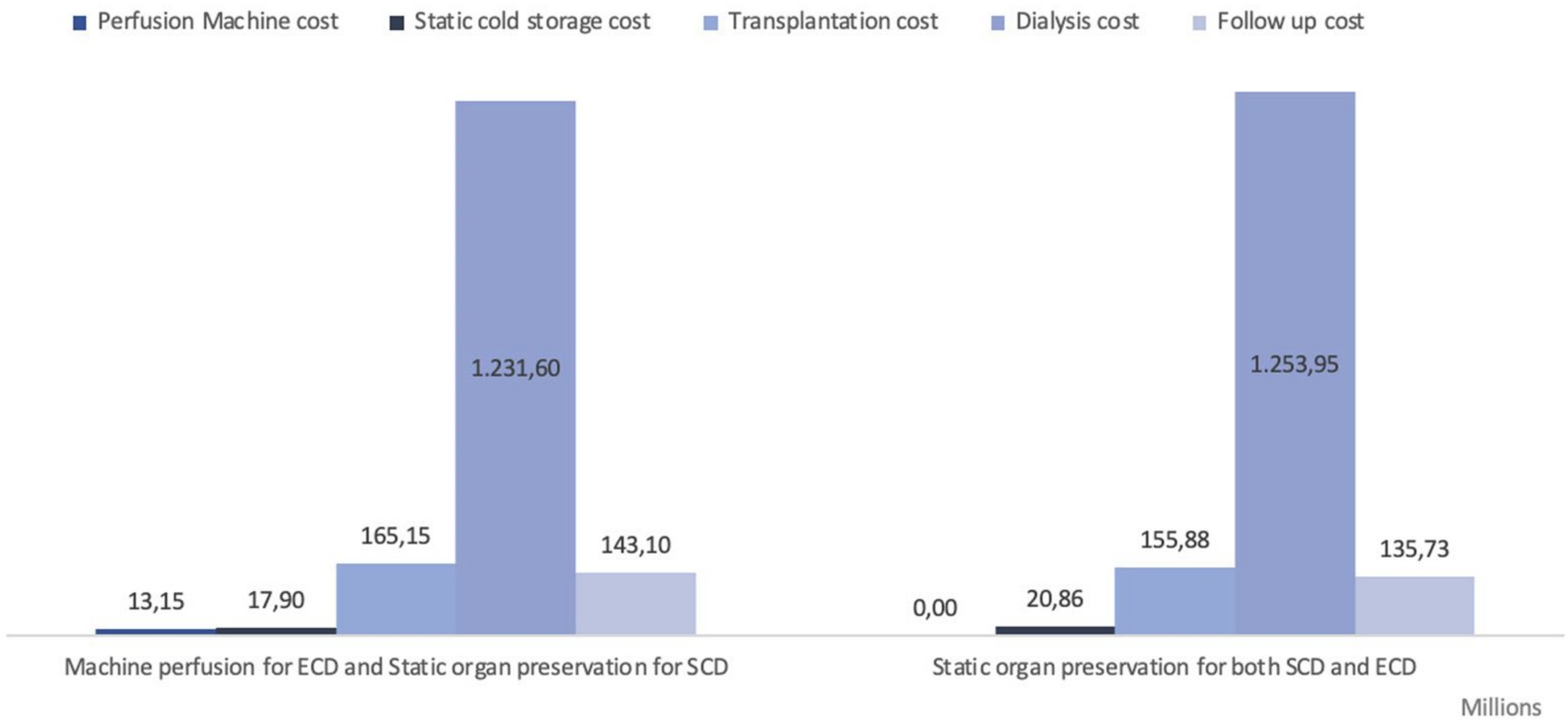

Perfusion Machine cost
Static cold storage cost
Transplantation cost
Dialysis cost
Follow up cost
13,15
17,90
165,15
1.231,60
143,10
0,00
20,86
155,88
1.253,95
135,73
Machine perfusion for ECD and Static organ preservation for SCD
Static organ preservation for both SCD and ECD
Millions

Figure S2

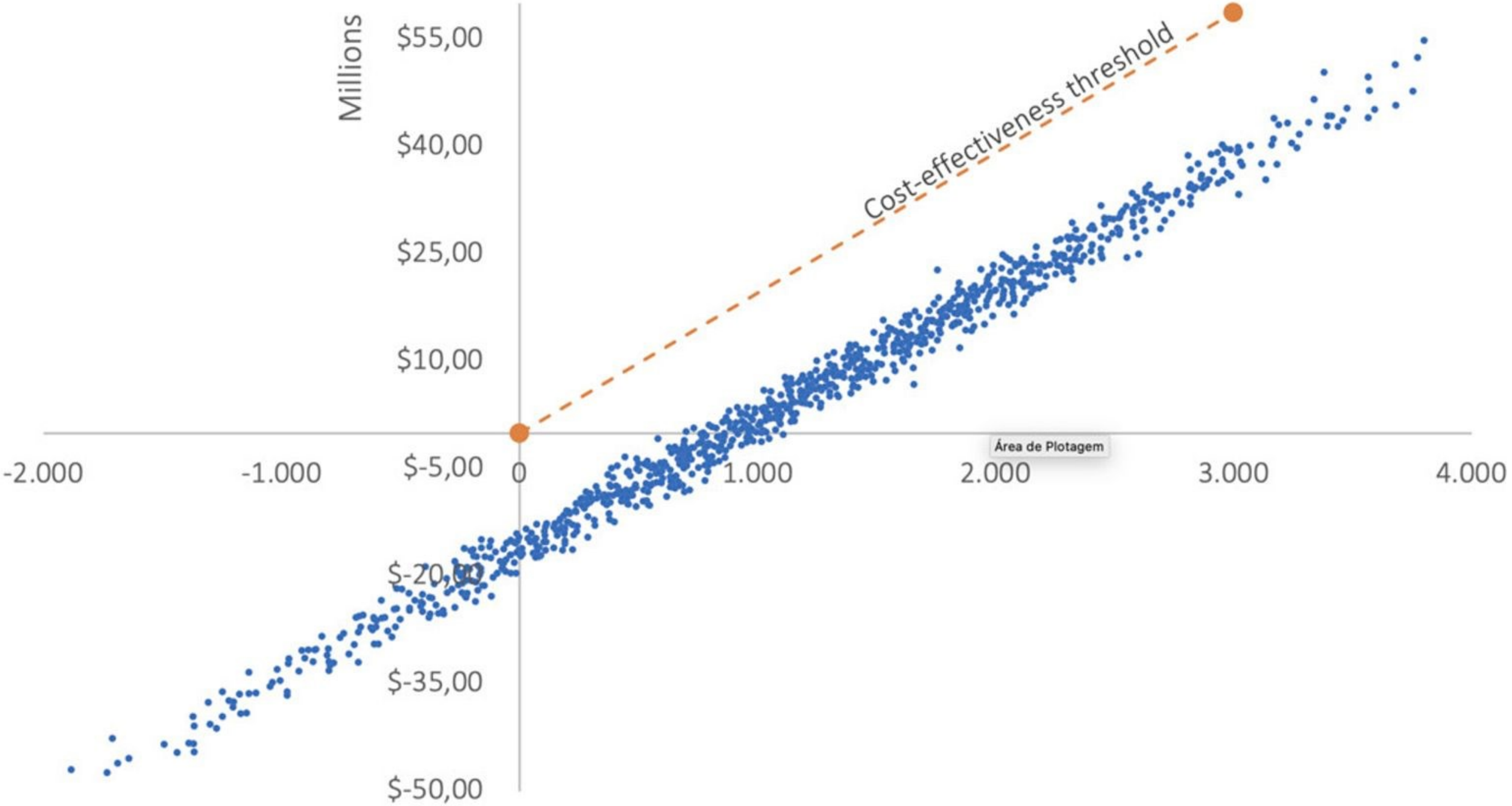
Millions
$55,00
$40,00
$25,00
$10,00
$-5,00
$-20,00
$-35,00
$-50,00
-2.000
-1.000
0
1.000
2.000
3.000
4.000
Cost-effectiveness threshold
Área de Plotagem

Figure S3

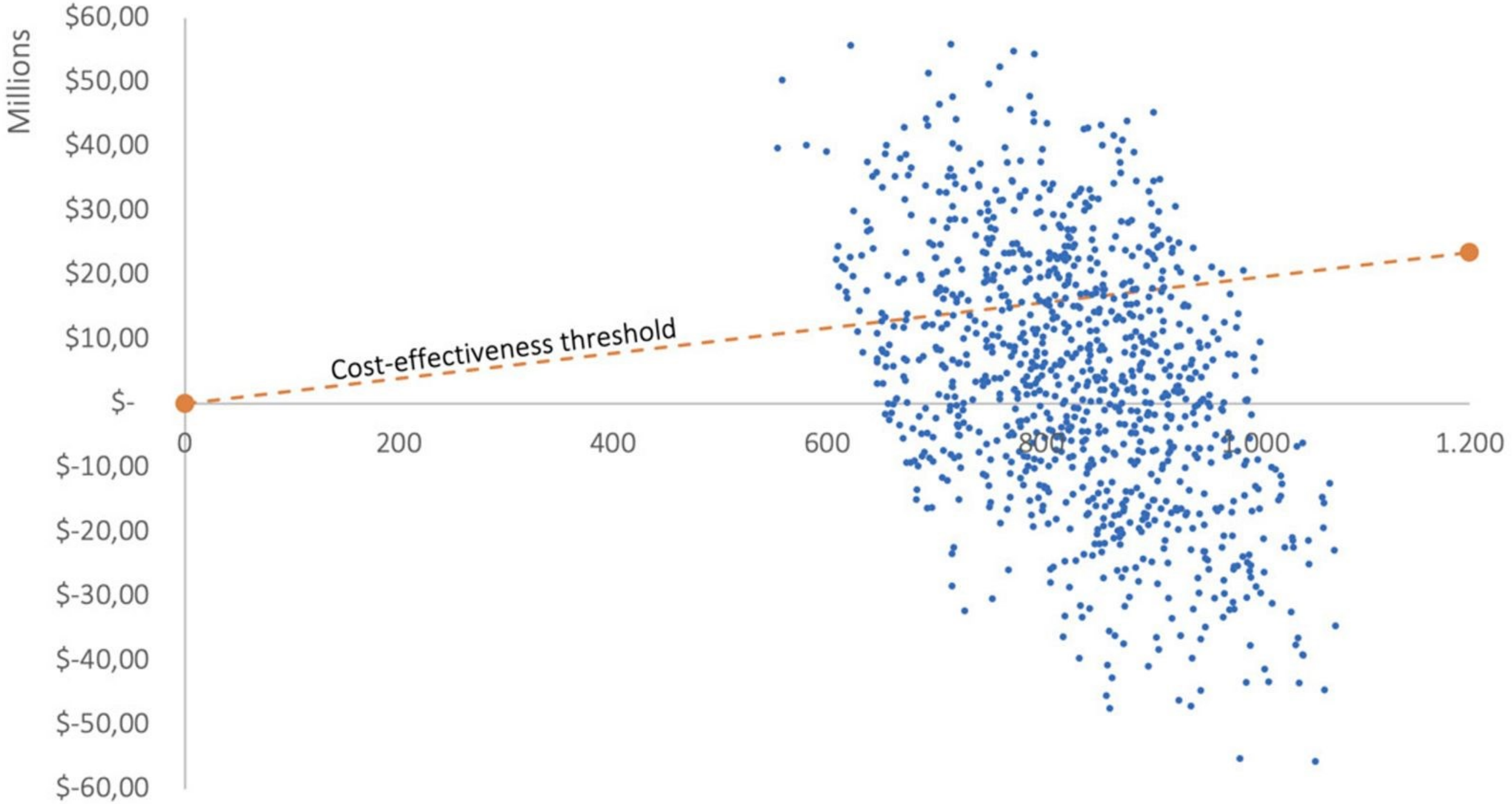

Millions
$60,00
$50,00
$40,00
$30,00
$20,00
$10,00
$-
$-10,00
$-20,00
$-30,00
$-40,00
$-50,00
$-60,00
0
200
400
600
800
1.000
1.200
Cost-effectiveness threshold

Figure S4

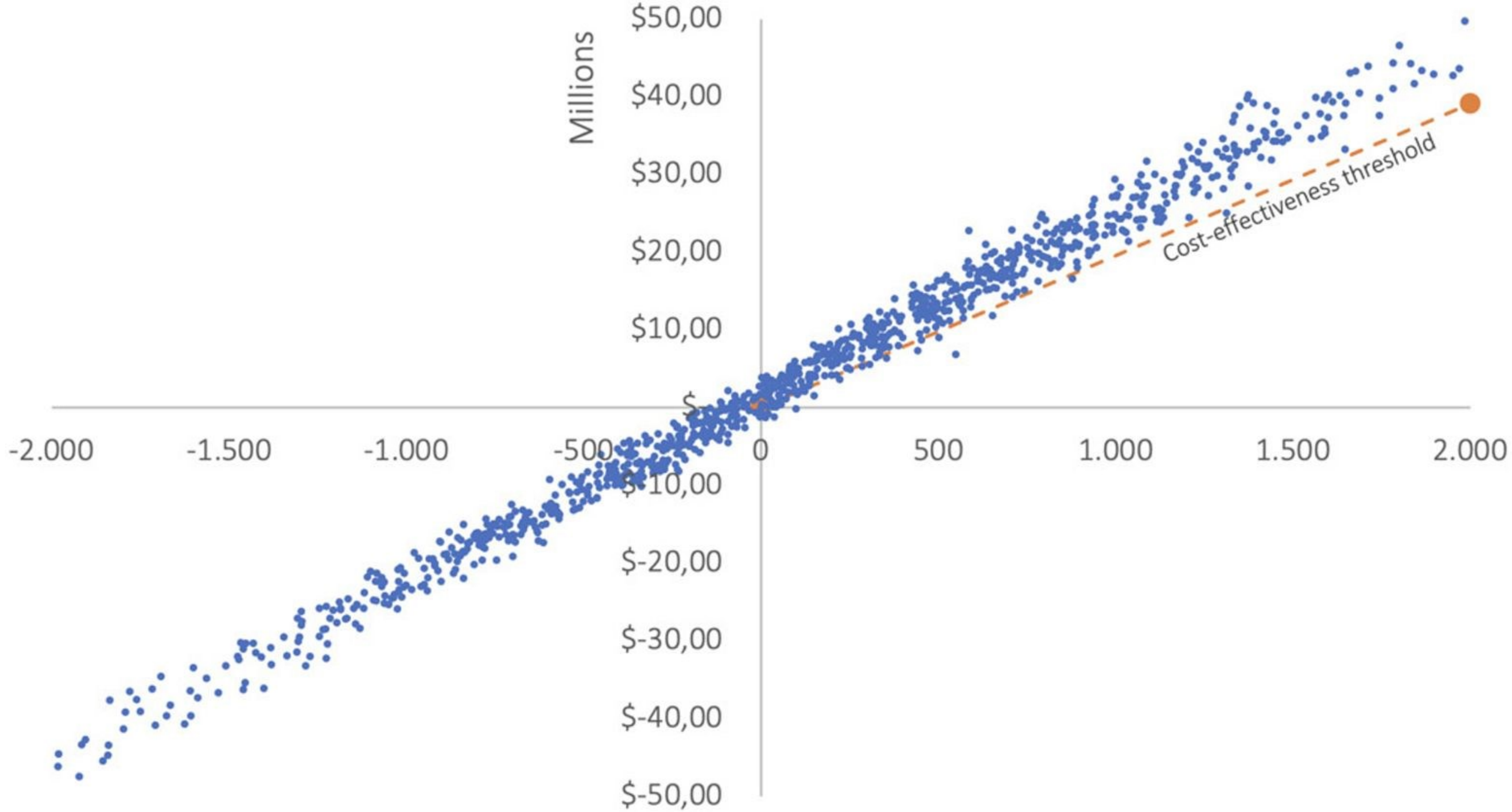

Millions
$50,00
$40,00
$30,00
$20,00
$10,00
$-
$-10,00
$-20,00
$-30,00
$-40,00
$-50,00
-2.000
-1.500
-1.000
-500
0
500
1.000
1.500
2.000
Cost-effectiveness threshold

FIGURE LEGEND

Figure S1. Distribution of costs according to procedures.

Figure S2. Probabilistic sensitivity analysis of cost-effectiveness per transplant performed.

Figure S3. Probabilistic sensitivity analysis of the cost-effectiveness per dialysis avoided.

Figure S4. Probabilistic sensitivity analysis of the cost-effectiveness per death avoided.
